# Convolutional Neural Networks for the Segmentation of Microcalcification in Mammography Imaging

**DOI:** 10.1155/2019/9360941

**Published:** 2019-04-09

**Authors:** Gabriele Valvano, Gianmarco Santini, Nicola Martini, Andrea Ripoli, Chiara Iacconi, Dante Chiappino, Daniele Della Latta

**Affiliations:** ^1^IMT School for Advanced Studies Lucca, Lucca, Italy; ^2^Imaging Department, Fondazione Gabriele Monasterio, Massa, Italy; ^3^Azienda USL Toscana Nord Ovest (ATNO), Carrara, Italy

## Abstract

Cluster of microcalcifications can be an early sign of breast cancer. In this paper, we propose a novel approach based on convolutional neural networks for the detection and segmentation of microcalcification clusters. In this work, we used 283 mammograms to train and validate our model, obtaining an accuracy of 99.99% on microcalcification detection and a false positive rate of 0.005%. Our results show how deep learning could be an effective tool to effectively support radiologists during mammograms examination.

## 1. Introduction

Breast cancer is one of the most common malignant neoplasms in the female population. The referral examination used for screening of breast cancer is mammography.

Mammography is a radiological procedure that uses a bundle of X photons to map the breast tissue attenuation. With the use of high-resolution detectors, it is possible to detect microstructures with a high atomic number in the breast. Among them, breast microcalcification (MC) can be an indicator for the diagnosis of breast cancer as it is the expression of cell necrosis.

In mammograms, microcalcifications appear as regions with high intensity compared to the local background, and they can vary in size and have shapes ranging from circular geometries to strongly irregular ones with sharp or soft contours.

The Breast Imaging Reporting and Data System (BIRADS) standardized the interpretation of MCs by defining a scale ranging from 2 (benign finding) to 5 (highly suspicious of malignancy) based on their shape, density, and distribution within the breast.

An important type of benign calcification that can be seen incidentally on mammography is breast arterial calcification (BAC), which seems to correlate with coronary calcification. Breast vascular calcifications are differentiated from malignant and ductal calcifications by size, morphology, and distribution and appear as linear “tram tracks” [1] of calcification along arterial walls with a winding rather than branching course on mammography.

Since there are studies [2] correlating the estimation of the patient cardiovascular disease (CVD) with the amount of calcium residing at vascular level inside the breast, the exact identification of the pixels belonging to a calcification can become crucial to assess possible outcomes for the future [3]. For this reason, the proposed system not only localizes MCs inside the tissue but also aims to provide the exact segmentation of these lesions.

Because of the variability of connective, glandular, and adipose tissue within the breast, microcalcifications are often difficult to find even for experienced operators. The heterogeneity of the breast tissue and projective image capture geometry implicate the impossibility to use a simple density threshold to automatically detect MCs. In addition, it is difficult to carry out the research by means of morphological filtering operations due to the large variability of their geometry.

Due to the intrinsic limitations of classical methods, with this work, we propose a system based on deep learning [4], demonstrating the potentialities of convolutional neural networks (CNNs) to effectively detect and segment breast microcalcifications to support radiologists in mammograms examination.

## 2. Related Works

In the literature, a wide range of algorithms has been proposed for the automatic detection of clusters of mammary calcifications, highlighting the importance of this task. The first attempts were mainly based on the spatial characteristics of these lesions; an example of that is the morphological system proposed by Zhao et al. [5]. Given the appearance of MCs as a locally high-intensity region, this work introduced a method based on the application of an adaptive thresholding operation on the mammogram, used to subsequently extract the lesions.

Subsequently, Wang and Karayiannis [6] proposed an approach employing the wavelet transform to emphasize local variations in contrast. The intuition behind wavelets usage resides in their ability to discriminate different frequency bands and the possibility to preserve signal details at different resolutions. In this context, microcalcifications correspond to high-frequency components in the image, and they can be detected by decomposing the mammograms into different frequency subbands and filtering out low frequency variations from the image.

Another proposal for the MCs detection pipeline is the multiresolutional analysis carried out by Netsch and Peitgen [7], where the detection of microcalcifications is based on the Laplacian scale-space representation of mammograms.

Later on, several papers proposed machine learning approaches to solve the task. Particularly, Edwards et al. [8] formulated the MCs detection task as a supervised-learning problem and employed a Bayesian neural network to detect true MCs among several candidates obtained by a preliminary analysis of the mammogram. A second machine learning approach is the one proposed by El-Naqa et al. [9], who investigated instead, the possibility to apply support vector machines to develop the detection algorithm.

Unfortunately, even if in some cases these methods were able to achieve a good sensitivity (i.e., in [9], a sensitivity of 94% was achieved, outperforming all the other methods tested by the authors), most of the previous approaches usually suffer from a high false positive rate. This weakness is a direct consequence of the great variability of the breast tissue which must be taken into account to avoid misses of true positives also in very different mammograms.

Due to intrinsic limitations of classical methods, recent years have seen an increasing interest in nonlinear approaches based on convolutional neural networks. Such tools allow the avoidance of hand-crafted features definition, providing at the same time both automatic feature extraction and evaluation for the task at hand.

In particular, Mordang et al. [10] used a CNN architecture inspired by the OxfordNet [11] and obtained state-of-the-art performance with one of the first studies employing deep learning tools. Later on, Wang and Yang [12] modelled the mammogram analysis as a bipartite procedure defining a model for MC detection consisting of two subnetworks: one operating on the local image window and the other on the surrounding image background; the two subnetworks extracted features from the mammogram in parallel and fed them together into a shared fully connected layer for classifying the input window as containing a MC or not.

Going further, in this paper, we propose a fast MC detection and segmentation procedure based on the usage of two CNNs: one to quickly detect the candidate region of interests (ROIs) and one to subsequently segment them. Later, our system identifies the clusters of MCs in the image.

Subsequent to the detection of calcification clusters, further development of this work might deal with the identification of potential cancers or identification of BAC for the CVD stratification. In this work, however, we focus only on the segmentation of MCs and the cluster detection, without making any clinical assessment of patient risk.

## 3. Materials and Methods

### 3.1. Model

Mammograms are high-resolution images, and they can correspond to big matrices (e.g., 4095 × 5625 pixels) which could be time expensive to analyse. For this reason, we developed a model consisting of two CNNs: we called the first CNN detector while the second one was called segmentator. Detector's role is detecting candidate ROIs to be analysed, while segmentator classifies every pixel inside the given ROI.

The process of suspect ROIs detection must be noncomputationally expensive because its role is to accelerate the processing of the whole mammographic image, and then we preprocessed input images using Otsu thresholding to detect background pixels and exclude them from further evaluation.

We implemented both neural networks in Python, using the open-source software library Tensorflow.

We chose a patch-based approach to process the input mammograms assuming the local information sufficient to classify such small and circumscribed regions. Moreover, by contrast with fully convolutional approaches, a patch-based approach allowed us to considerably increase the training set and easily perform a good data augmentation.

With this purpose in mind, we extracted squared patches with *N* × *N* dimension and their annotated labels from the available mammograms and segmentation masks. We associated *positive* labels to the patches fed to the detector when they contained a microcalcification inside and *negative* labels in the opposite case. On the other hand, we assigned *positive* or *negative* labels to the patches fed to the segmentator accordingly to their central pixel: being part of a calcification or not.

**Figure 1:**
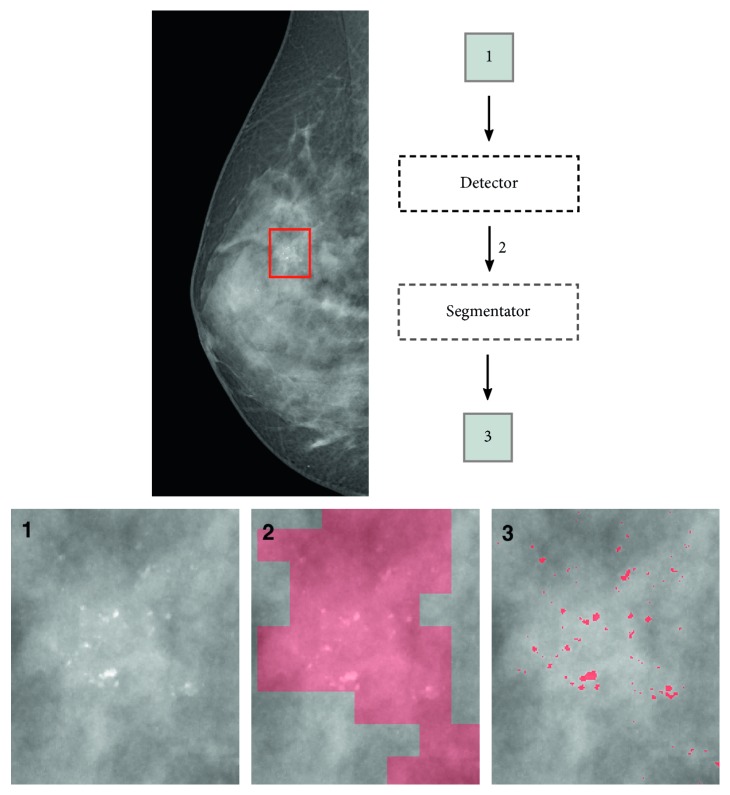
Example of breast segmentation process on a zoomed region in the mammogram (best viewed in color).

In the proposed model, once completed the training process of the networks and during test time, the input mammogram is patched at run-time with *N* × *N* windows overlapped by *N*/2 pixels on each direction. This overlapping area determines an increased redundancy while detecting the ROIs, limiting the false negative rate. Once the image has been patched, the detector classifies each of these *N* × *N* inputs with a binary label relating to the potential presence of MCs inside. Candidate ROIs are subsequently segmented by segmentator CNN, bringing to the creation of a MC binary mask (Figure 1).

The resulting mask is then analysed via a labelling algorithm in order to localize the position of every MC inside the matrix. Clusters are considered inside regions with more than 5 distinct MCs on cm^2^, as the radiological definition suggests [13].

### 3.2. Data

For our experiments, we used 283 mammography images with a resolution of 0.05 mm. Among these images, there are both natively digital mammograms and digitized images.

Every image is associated to the corresponding manual segmentation mask realized by a breast imaging radiologist. Since each segmentation mask consisted in a binary matrix, by classifying every pixel as a part of a MC or otherwise, we could utilize them to validate the obtained results.

In order to train our neural networks, the binary masks were also utilized to create the labels to be fed together with training samples to the CNNs, as ground truth.

We randomly chose 231 mammograms and the annotated labels to build the training set while 25 mammographic images were used to validate intermediate results and compare different networks architectures. The remaining 27 images were taken apart to build the test set and measure the final performances.

### 3.3. Construction of Training, Validation, and Test Set

We experimented different values of patch dimension *N*. The final dimension was chosen as a trade-off between computation burden and maximal information provided to CNN.

**Figure 2:**
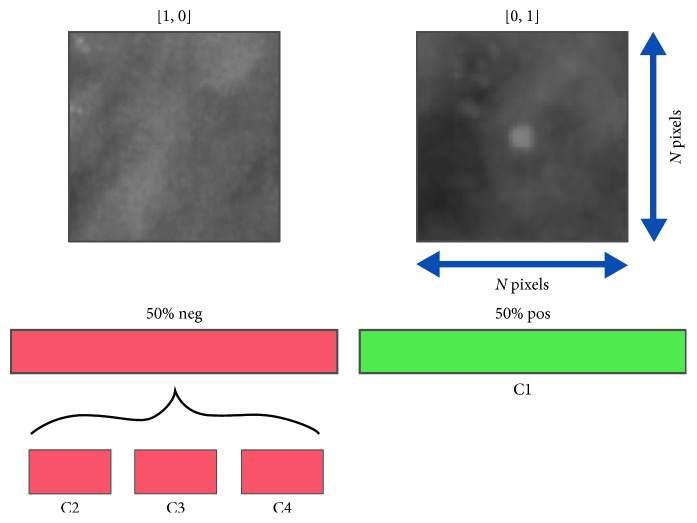
Example of patches and their subdivision in 4 different classes.

We paid particular attention to collect samples for training, validation, and test set, trying to make sure that the networks could always see as many input typologies as possible. For this reason, we contemplated 4 possible classes of patch (Figure 2) summarized in Table 1 and listed as follows:

- Class C1: patches whose central pixel belongs to a microcalcification
- Class C2: patches with MCs close to the center but with the central pixel not belonging to a calcification
- Class C3: cases where a calcification resides inside the patch but is located peripherally, and the central pixel does not belong to a MC
- Class C4: cases where no MC is present inside the patch

Since MCs are small circumscribed regions inside mammograms, certainly, class C4 contains the largest number of patches inside the database and class C1 is the less numerous class.

Moreover, we considered patches of class C2 as those containing calcifications in a range of 2 to 3 pixels from the center. This is a tricky class because as consequence of partial volume effect, MC border is frequently weakly defined and the classification of these pixels is often uncertain.

We organized the training set in a SQLite database to gather a customizable access to its samples during training. In particular, during the training process, we sampled patches belonging to each of these classes paying attention to feed the network with the same number of positive and negative samples, which means a good balance of input minibatches. We built each minibatch on the fly with random patches sampled from the database. Since this approach leads to always different minibatches, as a limit case, we could say we will not ever have exactly the same samples in two different minibatches, we believe it could improve the regularizing effect of batch normalization because it adds more randomicity to mean and variance inside minibatches. Moreover, we found this strategy useful to train networks with such a strongly unbalanced dataset.

In addition, we used data augmentation at training time to increase the dimension of the dataset with artificial samples, obtained randomly by rotating and flipping the images.

Patches for the validation set and test set were extracted from the 52 mammograms excluded from the training. Even in those cases, each set contained a balanced number of samples, considering the presence of each class inside.

### 3.4. Networks Architecture

**Figure 3:**
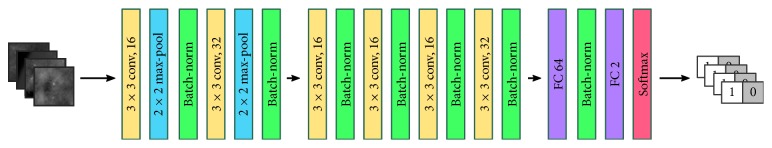
Neural network architecture implemented for segmentator and detector.

Both detector and segmentator CNN share the same architecture (Figure 3) consisting of 6 convolutional layers using 3 × 3 kernels and stride 1.

In particular, we tested the difference between the usage of a *same* convolution and a *valid* convolution for both the detector and the segmentator architectures. This means that we tested the possibility of applying the zero padding operation throughout the network layers in order to preserve the input dimension unchanged while going deeper. This trial aimed to understand if the *valid* convolution could invite the segmentator to gradually reduce the input patch to features concerning only its central regions (i.e., from 49 × 49 patches to 2 × 2 features inputs to the final fully connected layers) and help it during the classification task; on the other hand, since the detector should extract features from the whole input patch, we wondered if it could be favored by not reducing inputs through convolutions. In the results, we demonstrate the differences between these two approaches.

In the architecture, the first and the second convolutional layers are followed by a max-pooling layer with 2 × 2 kernel to reduce the computational burden and induce the network to extract more abstract representations of the data. Two fully connected layers of 64 and 2 hidden units close the architecture. We additionally used the drop-out strategy with 50% probability over the 64-units fully connected layer to limit network overfitting.

Exception made for the last fully connected layer, each one of the above layers is followed by a batch normalization layer, which we found gave a consistent speed up in the learning process, as suggested by Ioffe and Szegedy [14] and Santurkar et al. [15]. Finally, we used He initialization [16] to set the initial values for the weights and lessen their dependence on the initial state.

Neural networks often employ the softmax function to map the nonnormalized output to a probability distribution over predicted output classes. In line with the literature, we computed the posterior probability to belong to the *l*-th class given the vectorized input patch *X* and the network weight matrix *W* for each pixel *y*_*i*_ of the vectorized output matrix *Y* as

(1) $$\begin{array}{c} p\left(y_{i}=l\;|\;X\right)=\frac{e^{s_{l}}}{\sum_{k=1}^{K}e^{s_{k}}},\;l=1,\ldots ,K, \end{array}$$

where *s* is the score function defined as *s*=*f*(*X*, *W*), given *f*(·) the nonlinear function modelled by the neural net. With this formalism, *s* corresponds to the unnormalized log probabilities of the classes.

As common in modern CNNs, we trained the model employing Stochastic Gradient Descent with Adam optimizer [17] to minimize the categorical crossentropy:

(2) $$\begin{array}{c} H\left(y,\tilde{y}\right)=-\underset{j}{\sum }y_{j}\cdot \;\log\left({\tilde{y}}_{j}\right), \end{array}$$

where *y*_*j*_ is the ground truth label for the *j*-th class and ${\tilde{y}}_{j}$ is the network output over that class.

We trained each network with 256 sized minibatches. Moreover, we chose a learning rate of 1 × 10^−3^, but we manually halved this value when the loss plateaued in order to accelerate the convergence toward the minimum of the cost function.

We applied early stopping strategy, taking the last obtained model configuration before any evidence of overfitting on training set.

## 4. Results

Patch dimensions with side of 29, 39, and 49 pixels were tested. The following results relate to the usage of patch dimension *N*=49, which gave better performance and the employment of a convolution of type *same*.

We obtained a final accuracy of 98.22% for detector CNN and an accuracy of 97.47% for segmentator CNN when considered independently and tested on a balanced number of patches extracted from the test set images.

We did a more extensive analysis on the segmentation masks extracted from the entire mammograms. Since most classical methods suffer from a high false positive rate (FPR) in the MC detection process, we calculated the FPR obtained from our system. In particular, we analysed the binary masks obtained from the whole system (segmentator working on the preliminary ROIs identified by detector CNN on the entire mammograms), and we obtained an FPR of 0.005%. The final accuracy was 99.99% instead.

Table 2 shows the error rate obtained for each contemplated class and the overall accuracy obtained on test samples. In particular, the table presents the values obtained using a *valid* convolution approach and a *same* convolution approach for both the detector and segmentator.

**Figure 4:**
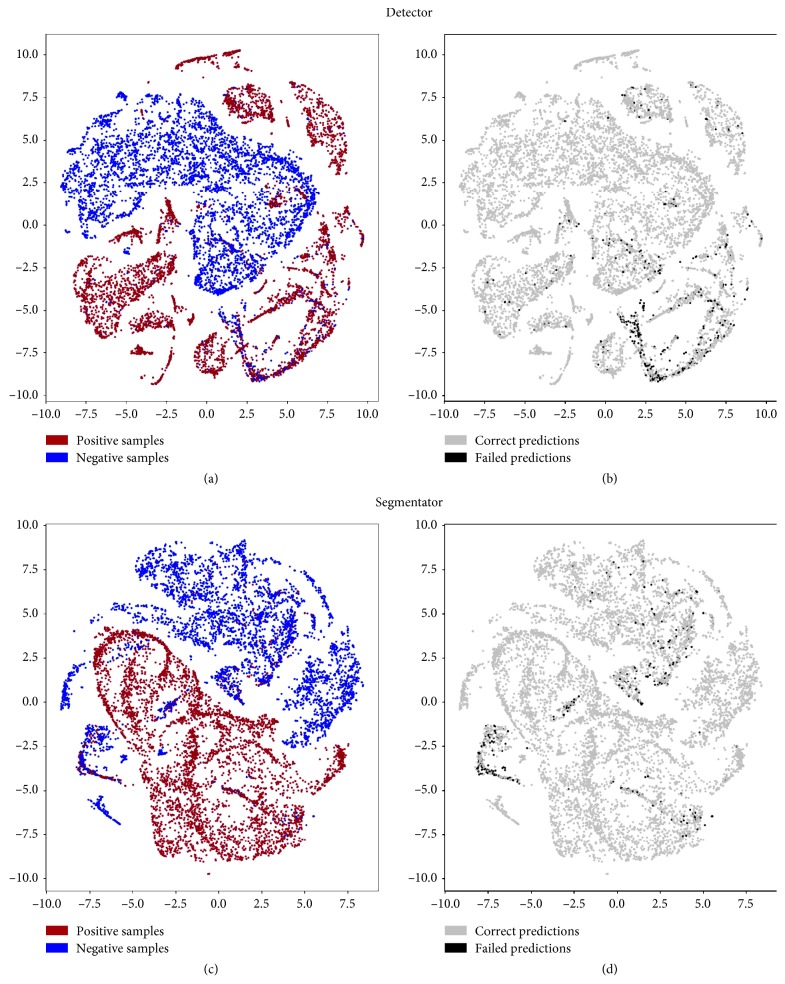
Latent feature spaces of the first fully connected layer projected on 2D plane for the detector and segmentator neural networks. (a, c) Projection of samples from the positive and negative classes. (b, d) Misclassified sample position in the bidimensional projected spaces (best viewed in color).

**Figure 5:**
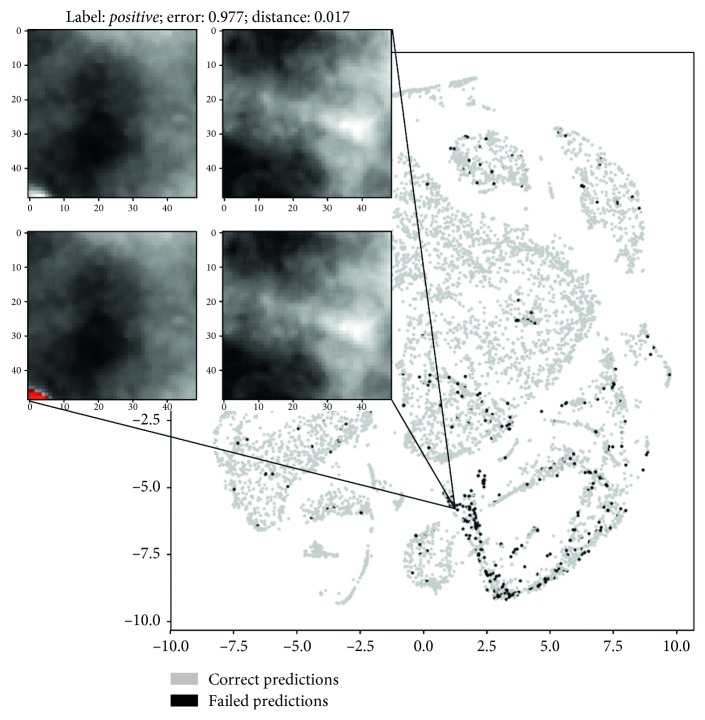
Example of detector failure case. The patch on the left represents the misclassified input sample containing a microcalcification, while the patch on the right is the (well classified) closest class C4 sample in the features space. Below, you can see the ground truth segmentations. Please note that the maximum possible error is equal to 1 and an error <0.5 means that the input patch is still correctly classified (best viewed in color).

**Figure 6:**
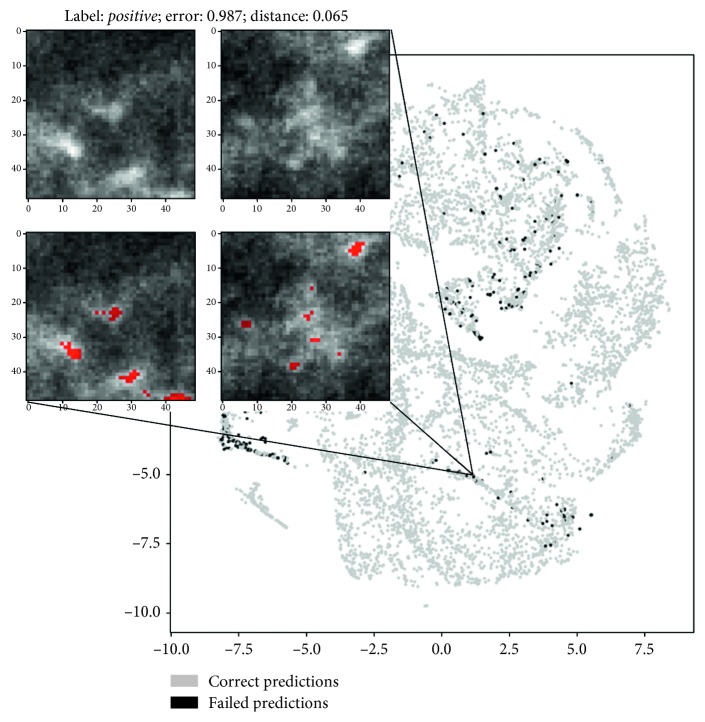
Example of segmentator failure case. The patch on the left represents the misclassified input sample containing a microcalcification, while the patch on the right is the (well classified) closest class C2 sample in the features space. Below, you can see the ground truth segmentations. Please note that the maximum possible error is equal to 1 and an error <0.5 means that the input patch is still correctly classified (best viewed in color).

To deepen our understanding of the network behavior, we also conducted an analysis of misclassified patches in the features domain using a nonlinear dimensionality reduction technique, namely, t-SNE [18], to project the feature vectors extracted by the layer preceding the final classifier (the output of FC 64) on two dimensions.  Figure 4 shows the 64 feature vectors projections on 2D planes for either segmentator or detector. Colors on the figures left represent the two input classes, while colors on the right represent right and wrong predictions operated by the CNNs. Figures 5 and 6 analyse more in depth the figures on right and wrong predictions, showing examples of misclassified patches and their nearest neighbors in the features domain (which we should expect to be similar to each other).

We tested the entire model on GPU GTX 970, and every mammogram was processed in roughly 20 seconds.

An example of input region segmented by the model is represented in Figure 1.

## 5. Discussion

From the results illustrated in Table 2, we could experiment how performing a convolution of type *same* instead of *valid* led to a relevant difference in both detector and segmentator performance over each class.

We believe these differences to be independent from stochastic oscillations of the cost function during the network training. Instead, this improvement can most probably be explained by the observation that using *same* convolution leads to larger inputs to the first fully connected layer and consequently to a larger number of weights at this level, resulting both in minor contextual information loss and larger model learning capability.

We consequently prefer the usage of a *same* convolution approach in both cases since it leads to better results and subsequently to a higher generalization capability of the model.

With regard to the gap in the overall performance between the segmentator and the detector, it can have many contributions and/or interpretations. There is obviously a stochastic component due to the optimization procedure; moreover, the task performed by the detector may be easier than the one carried out by the segmentator (i.e., the detector may be looking to the whole input patch rather than only on its central pixels, relying on other signal carriers).

Table 2 also outlines how the hardly classified classes mainly relate to limit cases between the assignment of *positive* or *negative* labels. We could assume class C2 to be a tricky class for the segmentator because, out of the partial volume effect (the partial volume effect can be defined as the loss of apparent activity in small objects or regions of the image because of the limited resolution of the imaging system), MC borders are often soft and hardly evaluated even by humans. On the other hand, it can be observed how class C3 achieves the worse performance during the detection process, which is probably due to the presence of patches with only a small number of peripheral pixels belonging to a MC (i.e., with only the one most external pixel in the corner of the patch) and, again, often belonging to MC border regions.

As a confirmation of all these hypotheses, we conducted an analysis of the misclassified patches both for detector and segmentator neural networks. In particular, we analysed extracted data representations from the penultimate fully connected layer using t-SNE.

We examined C2, C3, and C4 top misclassified patches from the segmentator and extracted the nearest neighbors in the features domain belonging to class C1 and vice versa. On the other hand, we examined C1, C2, and C3 top misclassified samples from the detector and corresponding nearest neighbor samples in the features domain which belonged to class C4 and vice versa.

An example of meaningful misclassified patches and their closest *positive* or *negative* samples in the latent space is illustrated in Figures 5 and 6. Visual analysis of results seems to confirm our preliminary interpretation of the errors.

By contrast, we highlight how Table 2 also points out that the error rate is maintained low in nonlimit cases, which is desirable. In fact, for the segmentator CNN, this means that even if the network fails classifying boundary pixels of a calcification, it generally recognizes its presence, achieving a good lesionwise accuracy when the lesion resides in the central region of the input patch. At the same time, the detector shows a good lesionwise accuracy when MCs are well visible in the input data, failing only with more peripheral lesions. In this context, we considered an overlap of half patch along each direction during the mammogram analysis to prevent MC misses during the detection process and consequently breaking down detector error rate on class C3 samples.

**Figure 7:**
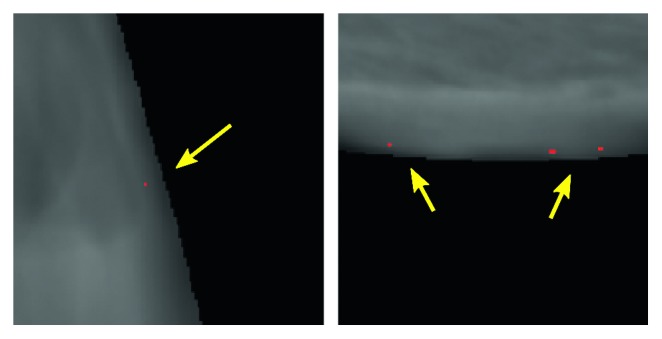
Example of false positives in correspondence of the transition region from the breast tissue to the background pixels (best viewed in color).

In addition, aside from tricky classes, an interesting fact pointed out by a qualitative analysis of the segmentation masks concerns a certain inclination of the model to make mistakes in correspondence of the transition region from the breast tissue to the background pixels. This is probably due to the fact that these regions usually relate to areas with strong contrast variation. An example of the phenomenon is illustrated in Figure 7.

Finally, this analysis highlighted how the major source of false positives seems to reside in the digitized images, where the presence of a greater quantity of widespread noise leads the network to commit a greater number of errors.

## 6. Conclusion

We propose a model to detect and segment breast microcalcifications within mammographic images. This model is composed of two consecutive blocks based on convolutional neural networks: the detector and the segmentator. Thanks to the preliminary analysis carried out by the first CNN, the computational burden is considerably reduced and the total segmentation process does not become time consuming.

Moreover, the quality of the achieved results suggests the potentialities of this tool to effectively support radiologists during mammograms examination, bringing aid during the nontrivial evaluation of uncertain regions and reducing the diagnosis time. This could be especially useful in the screening setting, where the large number of examinations could reduce the attention of the reader, to support diagnosis or to narrow differential diagnosis.

## Figures and Tables

**Table 1:** Summary of the patch classes.

MC position	Class C1	Class C2	Class C3	Class C4
Center	✓			
Close to the center		✓		
Periphery			✓	
Outside				✓

**Table 2:** The obtained test error rate for each class and the overall test accuracy for the detector CNN and the segmentator CNN, using both valid and same convolution.

	Class C1	Class C2	Class C3	Class C4	Overall test accuracy
Detector					
*Valid* architecture	0.19	1.22	**20.28**	1.69	96.04
*Same* architecture	0.09	0.47	**7.84**	2.09	**98.22**

Segmentator					
*Valid* architecture	1.85	**15.32**	0.34	0.55	96.37
*Same* architecture	1.57	**10.03**	0.24	0.20	**97.47**

## Data Availability

The data used to support the findings of this study are restricted by the ethical board committee in order to protect patient privacy.
